# Experimental Investigations on Shear Thickening Fluids as “Liquid Body Armors”: Non-Conventional Formulations for Ballistic Protection

**DOI:** 10.3390/polym16162305

**Published:** 2024-08-15

**Authors:** Florentina Alexe, Ciprian Sau, Ovidiu Iorga, Gabriela Toader, Aurel Diacon, Edina Rusen, Claudiu Lazaroaie, Raluca Elena Ginghina, Tudor Viorel Tiganescu, Mircea Teodorescu, Arcadie Sobetkii

**Affiliations:** 1 Research and Innovation Center for CBRN Defense and Ecology, 225 Olteniţei Ave., 041327 Bucharest, Romania; crinaalexe@yahoo.com (F.A.); ciprian.sau@nbce.ro (C.S.); claudiu@nbce.ro (C.L.); ginghinaraluca@gmail.com (R.E.G.); 2 Military Technical Academy “Ferdinand I”, 39-49 George Cosbuc Boulevard, 050141 Bucharest, Romania; aurel_diacon@yahoo.com (A.D.); viorel.tiganescu@mta.ro (T.V.T.); 3 Faculty of Chemical Engineering and Biotechnologies, National University of Science and Technology Politechnica Bucharest, 1-7 Gh. Polizu Street, 011061 Bucharest, Romaniamircea.teodorescu@upb.ro (M.T.); 4 SC MGM Star Construct SRL, 7 Pincota Street, 021784 Bucharest, Romania; sobetkii@yahoo.com

**Keywords:** shear thickening fluids, polymers, composites, ballistic protection, liquid armor

## Abstract

Shear thickening fluids (STFs) have garnered attention as potential enhancers of protective capabilities and for the optimization of Kevlar^®^ armor design. To assess the possible shear thickening properties and potential application in ballistic protection, ten formulations were developed by employing polyethylene glycol (PEG) or polypropylene glycol (PPG), along with fumed silica or Aerosil HDK^®^. Rheological characterization facilitated the identification of formulations displaying shear thickening behavior. The potential integration of the selected shear thickening fluids (STFs) into Kevlar^®^-based composites was investigated by studying the impact resistance of Kevlar^®^ soft armor structures. Also, high-velocity impact testing revealed that the distance between aramid layers plays a crucial role in the impact resistance effectiveness of Kevlar^®^–STF composite structures and that there is a very narrow domain between optimal and undesired scenarios in which STF could facilitate the penetration of Kevlar. The introduction of STF between the Kevlar sheets disrupted this packing and the energy absorption capacity of the material was not improved. Only one formulation (PEG400, Aerosil 27 wt.%) led to a less profound traumatic imprint and stopped the bullet when it was placed between layers no.1 and no.2 from a total of 11 layers of Kevlar XP. These experimental findings align with the modeling and simulation of Kevlar^®^–STF composites using Ansys simulation software (Ansys® AutoDyn 2022 R2).

## 1. Introduction

Threats from armed conflict are constantly increasing, so ballistic armor always needs improvement. The ongoing investigations in the field of body armor are focused on the development of cost-effective, lightweight, and wearable systems designed to offer enhanced protection, comfort, and environmental awareness for personnel [1]. Several materials, including wood, leather, wool, bronze, and steel, have been employed to protect the human body over the centuries [2,3,4]. However, one of the first materials that truly revolutionized ballistic defense, starting in the mid-1960s, was aramid fibers [4,5]. Nowadays, ballistic protection mainly utilizes aramid fibers (Kevlar^®^) and ultra-high molecular weight polyethylene (Spectra^®^ and Dyneema^®^) [1,4,6]. However, up to 50 layers of fabric are required to achieve the protection standards for common ballistic threats, which leads to a bulk and rigid product that limits the comfort of the armor, restricting its use primarily to torso protection [1]. To address this inconvenience, Gates Jr. proposed, in 1968, a fluid armor system [7] to reduce the number of layers required for ballistic protection and develop a fully flexible material capable of self-healing after the impact [4]. Thus, starting from this idea, another innovative solution, which was patented [2,8] in the early 2000s, involved the coating of ballistic protection panels with non-Newtonian shear thickening fluids (STFs) for weight reduction, strength reinforcement, and cost-effective manufacturing [8,9,10,11,12]. STF-based liquid body armor has a promising potential to offer increased protection and flexibility [13].

The STFs suitable for ballistic protection applications are formulations with non-Newtonian flow behavior, commonly observed in concentrated dispersions and characterized by a substantial, sometimes discontinuous increase in viscosity when higher shear stress is applied [1,14]. The nonlinear rheological behavior of these particle suspensions results from a microstructural rearrangement of the particles within the system due to the increased shear rate or shear stress applied [13]. Thus, a shear thinning or a shear thickening effect can appear, depending on the composition, shear stress, and shear rate used. Concentrated suspensions subjected to high shear rates frequently exhibit shear thickening behavior [13].

STFs generally comprise a liquid carrier and dispersed solid particles. The materials employed for obtaining STFs are typically synthetic or naturally occurring minerals and polymers [13]. Many carrier fluids have been reported, including water, ethylene glycol (EG), and polyethylene glycol (PEG) [4,13]. Due to their conjoined stability, high boiling point, polarity, and non-flammability, EG and PEG are the most widely used [13,15,16]. Colloidal silica particles [17,18] suspended in polyethylene glycol are the most common examples of STFs [19,20,21].

The overall nature of shear thickening is determined by the physical parameters of the suspended phase: phase volume, particle size distribution [22], and particle shape, as well as those of the suspending phase: molecular weight [23], viscosity, functional groups of coupling agents [24], and deformation patterns [13,25]. It has been widely reported that the size of the particles in a suspension influences its rheological properties [13,26,27,28]. The volume fraction of the discrete phase is one of the variables that has demonstrated the most significant influence over nonlinear rheological behavior in suspensions [13].

Two primary theories have been posited to elucidate the shear thickening phenomenon [29]. This occurrence involves an increase in the viscosity of a fluid in response to heightened shear or flow rates. The two theories have been advanced to offer a comprehensive understanding of the underlying mechanisms governing shear thickening behavior across diverse fluid types and under varying environmental conditions. One theory suggests that nearby particles cluster together to form ‘hydroclusters’, which increases the resistance to fluid motion [30,31,32,33,34,35,36]. The other theory proposes that there is a transition from an organized layered formation of particles to a disorganized state, resulting in increased viscosity. This theory is often referred to as the “order–disorder theory (ODT)” [15,37]. It has been reported that larger particles have an increasing effect on the thickening and critical shear rate. Additionally, the type of carrier fluid used has an impact on the overall shear thickening effect and critical shear rate. It has been observed that larger particles of fumed silica increase the thickening effect and critical shear rate. The choice of carrier fluid also influences shear thickening performance and the critical shear rate [38]. Moreover, the synchronous gradient thickening and flow of STFs play a crucial role in enhancing the material’s ability to bear loads and effectively diffuse stress. This phenomenon has significant implications for various applications in engineering and materials science [39]. Until now, one of the most important applications of STFs comprises ballistic protective clothing (also known as liquid body armor). Most “liquid body armors” described in the literature were developed by immersing a fibrous substrate or porous media in an STF [4,7,9,10,12]. Lee et al. [1] reported the ballistic penetration resistance of a woven Kevlar fabric moistened with an STF containing silica particles (450 nm) pre-dispersed in ethylene glycol. Rosen et al. [40] showed that a Kevlar–STF fabric target impacted at low velocity (lower than 144 m/s) performs better than neat Kevlar. Yet, significant fiber fracture occurred at impact speeds higher than 150 m/s, and the projectile penetrated the Kevlar–STF fabric [40]. Under low-velocity impacts, STF addition seems to provide benefits similar to increasing fabric yarn count [41,42,43,44,45,46]. STF addition primarily reduces the mobility of filaments and yarns in the impact zone [47]. Yet, optimizing the STF composites is always recommendable for higher impact velocities. Applications of “liquid body armor” still have numerous drawbacks, such as evaporation, sensitivity to humidity, carrier fluid leakage, and reduced air or moisture permeability [13], which affect armor performances and comfort characteristics. For instance, in the case of an STF-composite containing silica particles suspended in ethylene glycol (EG), it is very probable to experience EG evaporation. In recent years, significant efforts have been made to address this issue.

To overcome the limitations of STFs, this study focused on developing and characterizing various STF formulations and identifying the ones most suitable for “liquid body armor”. In addition, the STF formulations with the most favorable premises for ballistic protection applications were utilized for developing Kevlar–STF composites with the following configurations: multilayered structures comprising STF-impregnated Kevlar^®^ XP woven fabrics and “sandwich” layered structures consisting of one thicker layer of STF between aramid woven fabrics layers, and their performances were evaluated via specific ballistics tests.

The novelty of this study resides in the thorough investigations performed on the ten STF formulations comprising polyethylene glycol (with different average M_n_ of 200 Da or 400 Da) or polypropylene glycol (average M_n_ 400 Da) as the liquid carrier and different concentrations of silica as particulate fillers with distinct particle-size distributions and various configurations of Kevlar^®^–STF composites, which demonstrated that the distance between the aramid layers is crucial. The findings of this experimental study align with the modeling and simulation of Kevlar^®^–STF composites performed via Ansys simulations software.

## 2. Materials and Methods

### 2.1. Materials

*Materials for STF formulations preparation:* Fumed silica—SiO_2_ average diameter 0.2–0.3 μm (Sigma Aldrich, St. Louis, MO, USA), Aerosil (HDK^®^ N20 pyrogenic silica, average diameter 40 μm, Wacker Chemie AG, Munich, Germany), **PEG200** (Polyethylene glycol, average M_n_ 200 Da, Sigma Aldrich, St. Louis, MO, USA), **PEG400** (Polyethylene glycol—average M_n_ 400 Da, Sigma Aldrich, St. Louis, MO, USA), **PPG400** (Polypropylene glycol—average M_n_ 400 Da, Sigma Aldrich, St. Louis, MO, USA).

*Aramidic fabrics:* Kevlar^®^—DuPont, Wilmington, Delaware, United States: Kevlar XP (specific mass—500 g/m^2^), Kevlar–carbon fiber composite fabric (specific mass—165 g/m^2^), Twaron^®^—Teijin Aramid, Arnhem, The Netherlands: Twaron T730 WRT (specific mass—260 g/m^2^), Twaron LFT AT FLEX (specific mass—490 g/m^2^).

### 2.2. Methods

#### 2.2.1. STF Formulations Preparation

Various STF formulations were prepared by combining PEG200, PEG400, and PPG400 with fumed silica and pyrogenic silica according to the procedure described below. The correspondence between the sample codes and their composition is summarized in Table 1.

The micrometric solid filler was added under continuous stirring in the liquid phase. The stirring process was carried out in the first phase with the help of a mechanical stirrer (500 rpm) until the micrometric silica particles were incorporated. On the formulation preparation progression, it was observed that dispersion of the SiO_2_ particles is a prolonged process, varying from 24 to 48 h for dispersing a 10 wt.% SiO_2_, and up to 60 h for the dispersion of a 30 wt.% SiO_2_. After 60 h of mechanical stirring, homogenization was further facilitated by an ultrasonic processor for some formulations (20 and 30 wt.% SiO_2_). The resulting formulations showed that the PEG 400 polymer enabled a better dispersion of silica particles than PEG 200 and PPG 400. Thus, based on the quality of the dispersions obtained, ten of the formulations mentioned above were chosen for further experimental investigations. For three of the ten formulations (those marked with * in Table 1), ethyl alcohol was used to incorporate a larger amount of SiO_2_. Further, ethyl alcohol was removed by placing the STFs in an oven at 60 °C for three days.

#### 2.2.2. Rheological Investigations on the STF Formulations

The rheological investigations were performed on a Kinexus Pro rheometer (Malvern Instruments, Malvern, UK, software 1.60) with a Peltier temperature control unit, employing 40 mm parallel plates, with 1 mm gap, in rotational mode, at 25 °C. All viscosity measurements were assessed with a shear rate range varying from 10^−3^ to 10^3^ s^−1^.

#### 2.2.3. Analysis of STF Performances at Impact with a Free-Falling Blunt Object (Dynamic Loading at Moderate Impact Speed)

To determine the dynamic impact behavior at moderate speeds, the formulations were packed in polyethylene foil to obtain rectangular-shaped specimens (dimensions: 10 cm × 10 cm × 1.5 cm), as illustrated in Figure S1 from the Supporting Information file. This test method (based on ASTM D7136 [48]) involved the free fall of a blunt metallic object, of known mass, from a known height. The tested specimens were placed on ballistic clay, and the sharp object was allowed to fall freely on top of them. The deformation of the STF formulations (the polyethylene packed samples) upon impact and their imprint in the ballistic clay were evaluated. The mass of the blunt projectile used was 1.004 kg, and the height from which it was dropped was 1.5 m. The potential energy of the object at the launch height is transformed into kinetic energy upon impact; thus, by applying the energy conservation Equation (1), an impact energy value of 14.77 J was obtained [49].

(1) $$E=m_{o}\cdot g\cdot h=\frac{m_{o}\cdot v_{o}^{2}}{2}$$

where ***m_o_*** represents the mass of the falling blunt metallic object, in kg; ***g*** represents gravitational acceleration, in m/s^2^; ***h*** represents the launching height, in meters; and ***v_o_*** represents the speed of the falling object, in m/s.

#### 2.2.4. Assessment of Kinetic Energy Absorption at Impact with Standard Shrapnel

**Figure 1:**
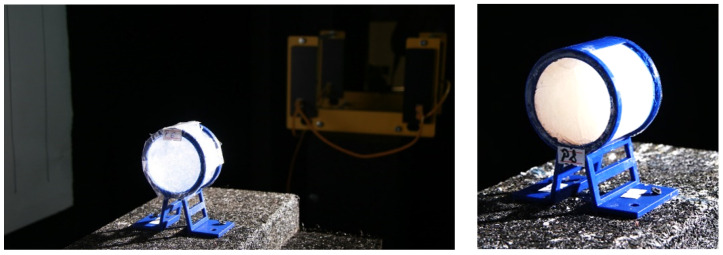
STF formulations loaded in a 3D-printed casing structure, utilized as targets for the assessment of kinetic energy absorption at impact with standard shrapnel.

The test method involves firing standard shrapnel [50] with a mass of 1.1 g through STF samples and evaluating the impactor’s kinetic energy loss following penetration of the analyzed material [51]. The STF formulations were loaded into 3D-printed casing structures built from polylactic acid, with transparent PVC film laterals, to form the specimens utilized as targets for this test. The casing structures were 3D printed using the Wanhao Duplicator I3 printer (Figure S2 from the Supporting Information file) and PLA (Polylactic Acid) filaments. An example of STF formulation loaded in a 3D-printed casing structure, utilized as targets for the assessment of kinetic energy absorption at impact with standard shrapnel, is illustrated in Figure 1.

The initial and final velocities (before and after the sample to be analyzed) were determined by two ballistic chronographs. First, a series of shots were performed without samples for the comparative validation of the velocities determined by the two chronographs. A correction factor of 9 m/s was applied to the velocities measured by the chronograph in front of the sample. Five specimens from each STF formulation were tested. Based on the experimentally determined velocities, the loss of kinetic energy was calculated for each STF formulation. The kinetic energy was calculated with Equation (2):

(2) $$E=\frac{m_{p}\cdot v_{p}^{2}}{2}$$

where ***m_p_*** represents the mass of the projectile, in kg, and ***v_p_*** represents the velocity of the projectile, in m/s.

#### 2.2.5. High-Velocity Impact Tests with 9 mm Bullet

This method of testing the resistance to high-velocity impacts consists of shooting various composite structures with ammunition of caliber 9 × 19 mm, with lead-core bullets, from a 5 m distance. The equipment and materials used for this test were a shooting stand equipped with a 9 mm caliber ballistic barrel and LASER pointer (Figure S3a from the Supporting information file), ballistic chronograph B462/HPI that ensures the automatic recording of bullet speed (Figure S3b from the Supporting information file), and 9 × 19 mm caliber ammunition, lead-core bullets which achieve velocities in the range of 340–440 m/s. Distinct types of aramid fabrics (Figures S4 and S5a–c, details in Materials section) with dimensions of 260 × 260 mm were utilized to create the composite structures for high-velocity impact tests (triplicate experiments).

SC0 was obtained by overlapping 11 sheets of Kevlar XP. SC1 was obtained by alternatively overlapping the two types of Kevlar sheets (one sheet of Kevlar–carbon fiber followed by one sheet of Kevlar XP, another sheet of Kevlar–carbon fiber, followed by another sheet of Kevlar XP, and so on). For SC2 and SC3, the same Kevlar sequencing was employed. The impregnated aramid–STF composites (Table 2) were obtained as follows: an appropriate amount of STF was deposited on each layer of backing material, over which another layer of backing material was placed and pressed with a rubber roller to allow the STF to interpenetrate with the backing fabric and remove the excess mix. This procedure was repeated for each layer until the final composite structure was obtained. SC-bk served as a blank specimen and was obtained by impregnating the Kevlar layers with PEG400.

The multilayered aramid–STF composite SC4 (Table 3) was obtained by overlapping 11 aramid layers and one STF layer (P4, packed in polyethylene, measuring 1 cm thickness). The honeycomb structure, which served as support for the STF formulation P10 in the SC7 (Table 3) configuration (combining 17 layers of Twaron T730 WRT and one layer of Twaron LFT AT FLEX, Figure S5e), was 3D-printed using PLA (Polylactic Acid) filaments. SC8 (Table 3) was obtained by overlapping two layers of Twaron T730 WRT, one STF layer P10 (1 cm thickness), five layers of Twaron T730 WRT, and one layer of Twaron LFT AT FLEX (Figure S5f). SC6 (Table 3) served as a control sample (Figure S5d).

#### 2.2.6. Modeling and Simulation of Kevlar/Shear Thickening Non-Newtonian Fluid Impact in Explicit Dynamics

For simulation, Ansys 2022 R2 was used in explicit dynamics. For the 3D CAD model, SolidWorks 2022 was used. The impactor was simulated as a 9 × 19 mm Parabellum bullet (copper jacket and lead core) as shown in Figure S6a. The copper jacket has a mass of 1.50 g, while the lead core has a mass of 6.56 g, giving the projectile a total mass of 8.06 g. The Kevlar/non-Newtonian “sandwich” structure, simulating the impregnated aramid–STF composites, was modelled as a disk of alternating Kevlar and fluid (Figure S6b), using 5 layers of Kevlar and 4 layers of non-Newtonian fluid, with the layer thickness of 2 mm for non-Newtonian fluid and 0.4 mm for Kevlar. For comparison, a secondary disk consisting of 5 layers of 0.4 mm Kevlar layers was also modelled, as presented in Figure S6c. The numerical model was prepared using Ansys Workbench. The material models used for simulation are presented in Tables S1 and S2.

## 3. Results

The STF formulations were prepared, characterized, and tested in different configurations to establish an optimal composition and configuration suitable to be employed in “liquid body armor” applications. To investigate the shear thickening abilities of the prepared formulations, rheological measurements were performed as the first step of this study, before testing the STF-based composites.

### 3.1. Rheological Investigations

**Figure 2:**
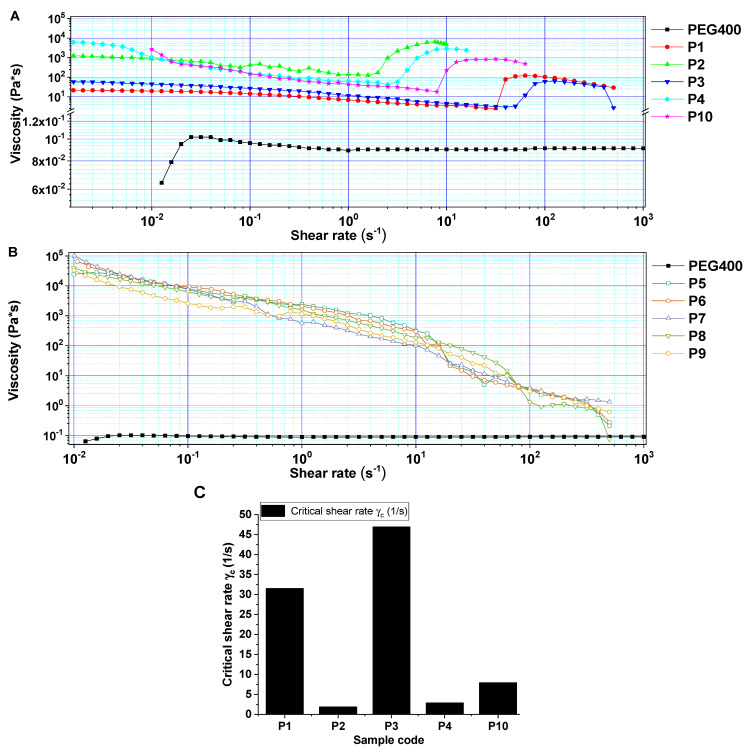
Rheological behavior vs. shear rate (**A**,**B**), critical shear rate values (**C**).

The rheological behavior of the STF formulations was tracked by studying the viscosity variation along with the increase in shear rate (Figure 2).

At low shear rates, most suspensions exhibited either Newtonian or shear thinning behavior. At higher shear rates, some of the formulations (P1, P2, P3, P4, and P10) entered a shear thickening regime that lasted no more than one order of magnitude before transitioning into another shear thinning region [14,52]. For P1–P4 and P10 formulations (PEG400), both types of filler (“fumed silica” and “pyrogenic silica”) induced a noticeable viscosity increase after reaching the critical shear rate γ_c_. The results are in agreement with the previous literature studies [12,19,28,53]. An elementary interpretation of the critical shear rate designates the threshold at which shear thickening commences and is a pivotal parameter for gaining an understanding of the rheological characteristics of STF. The critical shear rate (γ_c_) characterizes the transition from Newtonian to non-Newtonian behavior (shear thickening/shear thinning). A more precise definition of shear rate involves identifying the rate at which the tangent to the power law regime intersects the Newtonian viscosity line, which is commonly regarded as the critical shear rate value [54,55]. Shear thickening occurs easily when (γ_c_) is smaller [53]. As can be observed from Figure 2, the critical shear rate was lower when the concentration of nanofiller increased (P1, P3 vs. P4, P10), and it was less influenced by the type of silica employed. Sample 10 was the only one to maintain a high viscosity plateau for a longer time among all the samples that exhibited shear thickening behavior.

For formulations P5–P9, the viscosity decreases when increasing the shear rate, thus exhibiting a shear thinning behavior. The cause of this pseudo-plastic behavior may be the distinct unfolding of the PPG polymer chains in addition to the realignment and migration of the higher amount of suspended silica nanoparticles induced by the centrifugal force.

When analyzing the influence of two different polymeric matrices, namely PEG400 and PPG400, with the same molecular weight, for the same type and amount of nanofiller, on samples P1 and P3 (incorporating PEG400, shear thickening behavior) and P5 and P6 (incorporating PPG400, shear thinning behavior), it can be concluded that the slight difference in hydrophobicity introduced by the presence of methyl groups on the PPG400 chains may lead to fewer interactions between the polymeric chains and the silanol groups on the surface of the nanoparticles, resulting in a continuous decrease of viscosity when shear rate increases.

Moreover, due to the steric interactions, the methyl groups delay the polymeric chain accommodation [56], which may lead to supplementary entanglements, resulting in overall increased viscosity and difficulty in rearranging themselves under low to medium shear rates, thus obtaining no shear thickening effect. In contrast, for PEG400-containing samples (P1, P2, P3, P4, and P10), regardless of the type of silica nanofiller employed (in the concentration range of 20–30 wt.%), the shear thickening behavior was observable, probably due an easier flow of the polymer which resulted in an easier particle rearrangement, leading to a shear thickening effect. To summarize the results, in comparison to the PEG matrix, the PPG400 matrix does not offer any noticeable improvement in terms of shear thickening performances. Following the rheological measurements, it was determined that the formulations including PPG400 did not exhibit the desired properties for the intended application and were thus not pursued further.

Furthermore, integrating a larger amount of silicon oxide by adding ethyl alcohol led to clearly inferior results to those obtained by utilizing the PEG 400 matrix (without ethyl alcohol) and a lower amount of Aerosil.

### 3.2. Analysis of STF Performances at Impact with a Free-Falling Blunt Object (Dynamic Loading at Moderate Impact Speed)

The traumatic imprint resulting from the impact in the experimental conditions was smaller for the samples incorporating micronic nanofillers than in the case of the neat polymer matrices (Figure S1). The measured traumatic depths ranged between 15 mm and 27 mm for the STF formulations P1–P5 (Figure S1). Only the P10 sample (PEG 400 incorporating 27% Aerosil) exhibited a distinct behavior in terms of shear thickening, manifesting an increased viscosity upon dynamic impact with a blunt object, leading to a minimal imprint in the ballistic clay (Figure S1). This could be correlated to an optimal particle weight fraction that resulted in an increased shear thickening ratio [56]. Inversely, when the weight fraction outdistances the optimal level, the formation of particle clusters can occur, ultimately leading to the complete disappearance of the shear thickening effect. This could be attributed to the depletion of lubrication within the STF.

Based on the initial rheology assessment and the impact test with a free-falling blunt object, specific ballistics tests were further conducted to investigate the behavior of neat shear thickening fluids (STFs) or STFs in different aramid-based composite configurations.

### 3.3. Assessment of Kinetic Energy Absorption at Impact with Standard Shrapnel

**Figure 3:**
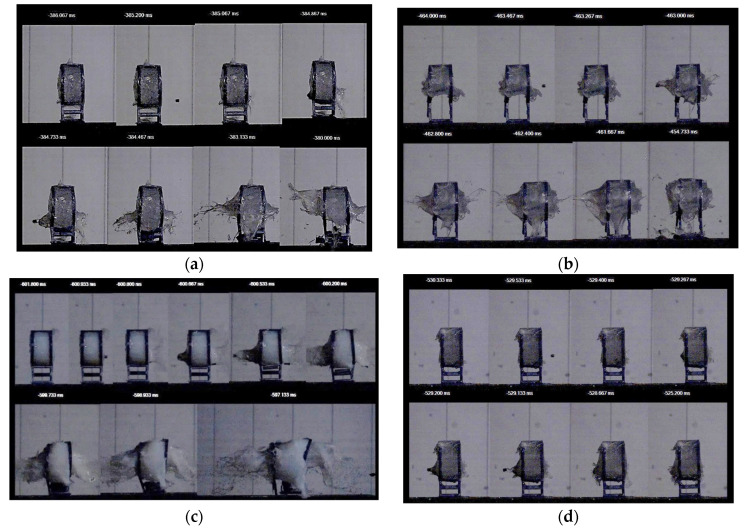
Exemplification of captures from STF impact with standard shrapnel. (**a**) P5; (**b**) P6; (**c**) P7; (**d**) P10.

Figure 3a–d depicts the behavior of several of the STF formulations upon impact with standard shrapnel (recorded with an ultra-high-speed camera). At a layer thickness of 35 mm, the investigated STF formulations achieved kinetic energy absorption percentages ranging from 45% to 60%, at values ranging from approximately 20 J to 30 J. The P10 sample provided the best results in terms of energy absorption and standard shrapnel impact behavior.

**Figure 4:**
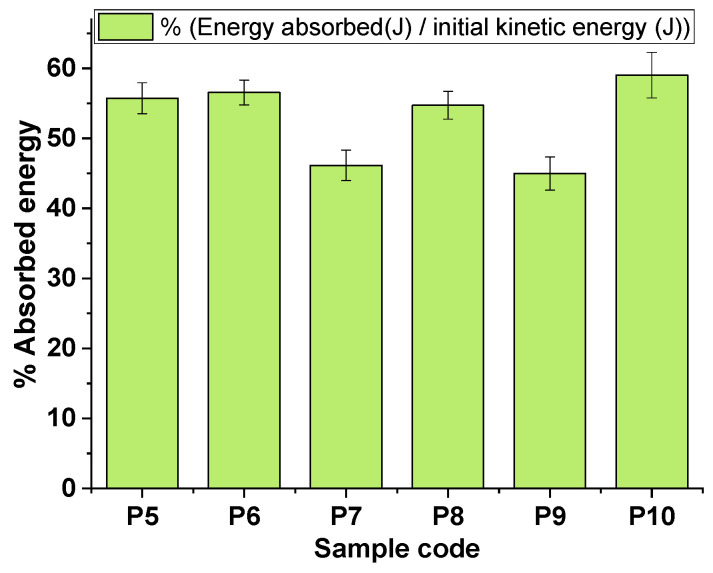
The percentual representation of the energy absorbed.

Figure 4 and Table 4 comparatively illustrate the energy absorption capacity of the STF formulations. It was observed that the energy absorption values were not influenced by the type of nanoparticle present in the STF. The concentration of the nanofiller and the nature of the carrier fluid had a more visible impact on the absorbed energy. In the same testing conditions, samples S7, S8, and S9, containing the same amount of Aerosil (40 wt.%.), registered distinct absorption energies, PEG400 (24.70 J) > PEG200 (21.78 J) > PPG400 (20.09 J), probably due to the distinct hydrogen bonding established between the polymeric matrix (PEG400, PEG200, or PPG400) and the silanol groups from the nanoparticles [57,58], thus leading to a different arrangement and flow of the polymer chains inside the STF when external stress was applied. The amount of H bonding in the solvation layer surrounding the silica may potentially lead to an increase in interparticle spacing [56], resulting in an improved deflocculation and enhanced interparticle repulsion forces. It is crucial to note that an elevated weight fraction of silica nanoparticles may prompt agglomeration, thus potentially impacting the performance. However, sample P10, which contained a smaller amount of silica nanoparticles, exhibited an optimal weight fraction of fumed silica, resulting in a more pronounced shear thickening effect and higher energy absorption. According to these findings, it may be advisable to consider a lower weight fraction of silica nanoparticles to prevent agglomeration and improve performance. P10 exhibited an exceptional ability to attenuate shrapnel, owing to an optimal amplification in frictional interaction.

The information collected, starting from the production step of the STF formulations and continuing with the investigation of their rheological properties, the evaluation of their behavior under dynamic impact with free-falling blunt objects, and the assessment of their energy absorption capacity at impact with standard shrapnel, were altogether taken into consideration in the development of the impregnated/multilayered STF-based aramid composite structures designed. This study further investigated the behavior of these composite structures with distinct configurations under dynamic stresses at high speeds.

### 3.4. High-Velocity Impact Tests with 9 mm Bullet

It is of utmost importance that, in the event of a ballistic impact, the projectile is effectively halted. Furthermore, the penetration depth into a clay witness backing the armor must not exceed 1.73 inches (44 mm) [1].

A wearer could suffer severe physical trauma if the penetration depth is greater than this value. When employing STF–aramid composites as “liquid armor”, the topology of the pore space causes a wide range of flow velocities and shear rates when non-Newtonian fluids flow through porous media [59]. After careful consideration, it was obvious that conducting high-velocity impact tests is essential to establish the most effective STF–aramid configuration for soft armor applications. The concept pursued in this study was to create a flexible composite structure for ballistic protection purposes, incorporating a high-strength material (aramid fibers) and a shear thickening fluid, and distinct configurations were tested to establish an optimal combination of these materials.

For the high-velocity tests, four types of aramid fabrics (details in Materials section), were utilized for obtaining the multilayered ballistic packages. Subsequently, two methods were utilized for the integration of STFs with these fabrics. The first approach involved impregnation of the fabrics with STF, while the second method utilized a singular, thicker layer of STF. The projectile initial velocity and initial kinetic energy are listed in Table 5.

**Figure 5:**
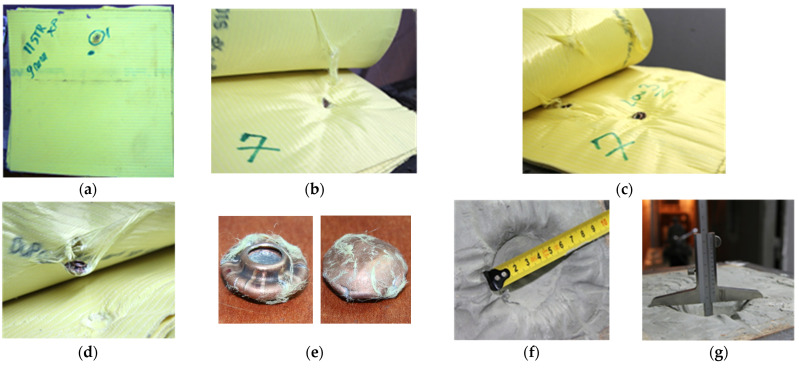
SC0: (**a**) 11 layers of Kevlar XP front view; (**b**–**d**) bullet stopped between 6th and 7th layers; (**e**) bullet recovered between Kevlar XP layers 6–7; (**f**,**g**) measuring the traumatic imprint in the ballistic clay.

SC0 was employed as a reference sample; therefore, when employing these 11 layers of Kevlar XP (which according to NIJ standards [60]) ensure an IIIA level of protection), the bullet was stopped and trapped between layers 6–7, and the traumatic imprint in the ballistic clay measured 36 mm (±2 mm) in depth and approximately 55–60 mm in diameter, as can be observed from Figure 5.

**Figure 6:**
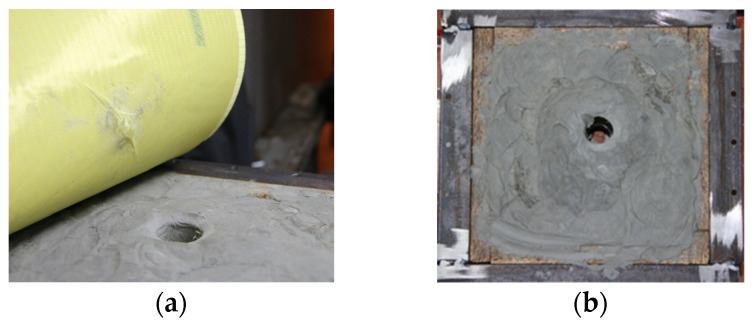
SC 1—(**a**,**b**) Total penetration of the Kevlar–carbon nanofiber interwoven configuration.

Kevlar–carbon fiber was chosen as a possible interlayer material due to its higher flexibility and its lower specific mass (Kevlar with carbon fibers—approx. 165.6 g/m^2^ vs. Kevlar XP—approx. 500 g/m^2^). Therefore, the SC1 structure included, in the following order, two layers of Kevlar XP, three layers of Kevlar–carbon fiber, one layer of Kevlar XP, three layers of Kevlar–carbon fiber, and two layers of Kevlar XP (Figure S5a–c). SC1 did not withstand the impact with the 9 × 19 mm caliber ammunition achieving total penetration (TP) of the composite structure (Figure 6).

**Figure 7:**
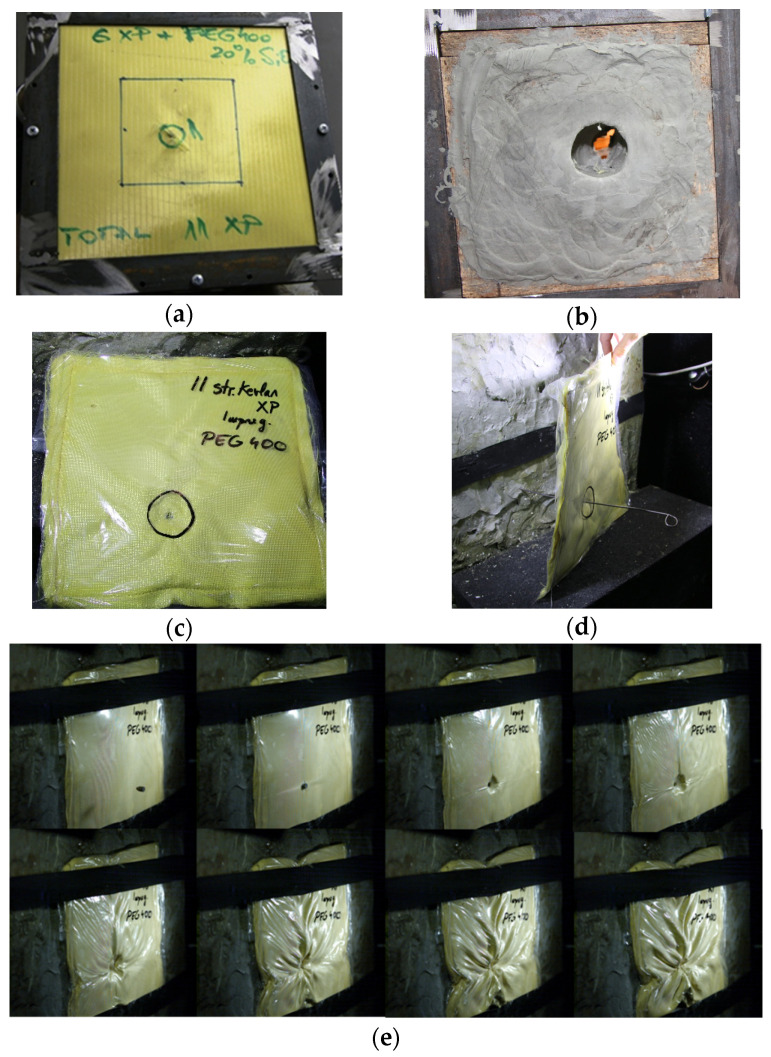
Total penetration of the impregnated aramid–composite configurations; (**a**,**b**)—SC 2 (shear thickening fluid—P1); (**c**–**e**)—SC-bk—P-bk (PEG400 only).

For SC2 composite, six out of the total of eleven layers of Kevlar XP were impregnated with P1 (PEG400, 20% fumed silica). For SC-bk, six out of the total of eleven layers of Kevlar XP were impregnated with P-bk (PEG400 only). In comparison with SC0 (11 neat layers of Kevlar), SC2 (eleven layers of Kevlar, of which six were impregnated with STF), and SC-bk (eleven layers of Kevlar, of which six were impregnated with PEG400) did not resist to the impact with the ammunition, achieving total penetration (TP) of the composite structure (Figure 7a–e). It can be confirmed that in this case, the ballistic resistance of the 11 layers of Kevlar XP was significantly decreased by the shear thickening formulations or by PEG400 when they were impregnated into six layers of aramid fibers. A similar behavior was observed for SC3, which also registered a total penetration of the composite.

To summarize the results obtained for all the STF impregnated—aramid composites tested, it can be affirmed that the impregnation of Kevlar layers with the STF formulations containing PEG 200, PEG 400, or PPG 400 and micrometric fumed silica or Aerosil^®^ (concentrations ranging between 20 wt.% and 40 wt.%) did not improve their ballistic protection performance. Moreover, the presence of STF reduced the performance of Kevlar XP in the impregnated configurations tested. Therefore, the next stage of this research was to investigate whether adding thicker layers of STF formulations in different configurations (detailed below, composition in Table 3) could improve ballistic protection.

**Figure 8:**
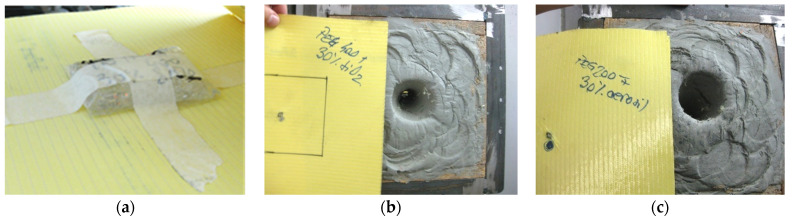
(**a**) 11 layers of Kevlar XP in SC4 or SC5 configuration; (**b**) Total penetration of SC4 structure; (**c**) Total penetration of SC5 structure.

**Figure 9:**
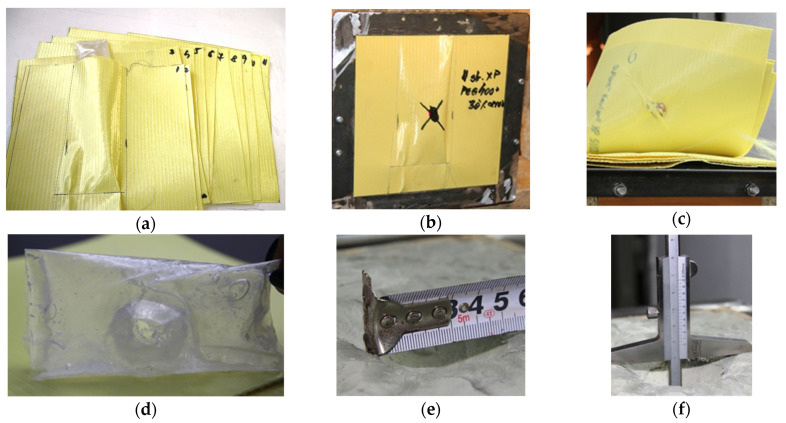
SC 6—(**a**,**b**) P4 placed between layers no.1 and no.2 from the total of 11 layers of Kevlar XP; (**c**) bullet stopped in layer no.6; (**d**) P4 aspect after the experiment; (**e**,**f**) measuring the traumatic imprint in the ballistic clay.

STF formulations measuring 1 cm thickness were utilized in two distinct configurations: (i) STF packed in polyethylene directly facing the bullet, as shown in Figure 8a (SC4 comprised P2 formulation—PEG400, fumed silica 30 wt.%; SC5 comprised P4 formulation—PEG400, Aerosil 30 wt.%)) and (ii) STF packed in polyethylene covered with one layer of Kevlar XP (SC6, which comprised only P4 formulation—PEG400, Aerosil 30 wt.%), Figure 9a.

In contrast with SC0, as can be observed from the images in Figure 8b,c, adding the P2 and P4 formulations to the 11 layers of Kevlar XP, on the direction facing the bullet, led to total penetration in both cases. However, an interesting aspect can be noticed when comparing the results for SC4 and SC5, which differ only in the type of STF used in front of the Kevlar: a higher critical shear rate (obtained for P4, used for SC5) leads to a slight increase in the absorbed energy.

Unexpectedly, when P4 was placed between layers no.1 and no.2 from the total of 11 layers of Kevlar XP, as shown in Figure 9a, the bullet was stopped and entrapped in layer no.6 of Kevlar XP. Moreover, the traumatic imprint measured ~30 mm in depth and ~50 mm in diameter. These dimensions obtained for SC6 are smaller than the ones measured for SC0, confirming the positive influence on the ballistic protection performances of P4 in the SC6 configuration.

The results suggest that the use of STF with the same number of Kevlar layers may frequently lead to total penetration. Nevertheless, it is important to note that there is only a slight difference between these undesirable cases and the ideal scenarios where the traumatic imprint is minimal and the bullet is stopped in the first five-to-six layers of Kevlar, as shown in Figure 9. An explanation for this behavior in the SC6 configuration could be related to the velocity of the bullet when reaching the STF layer, which is lower when the STF package is covered by a Kevlar layer. Thus, it is possible that in this case, the shear rate might have been in the shear thickening range, which did not happen for SC4 or SC5 if this optimal range was surpassed due to a higher velocity of the bullet when facing the STF layer. Thus, for SC4 and SC5 a shear thinning behavior after the thickening is confirmed during the high-velocity impact tests. Upon correlating the rheological findings with the results obtained from impact experiments, we have identified two potential mechanisms contributing to the heightened energy dissipation in the presence of STF: (a) viscous energy dissipation, ascribed to the elevated viscosity of STF at increased shear rates, and (b) augmented inter-yarn friction due to the presumed deposition of nanoparticles on the yarn/filament surface during impact. These observations are consistent with analogous studies found in the literature [61]. The kinetic energy is primarily absorbed through viscous dissipation, with a smaller amount in the form of frictional attenuation [61,62]. In concentrated suspensions, strong shear thickening is primarily caused by the frictional contact forces. In STF, the formation of silica clusters is influenced by a balance of hydrodynamic, thermal, steric, and frictional forces [63,64]. In many systems, shear thickening is the stress-induced change in particle flow from a lubricated, near-contacting flow to a frictionally contacting network of particles [64]. Using STFs with larger particle sizes, higher particle concentration, and higher molecular weight of the dispersion medium can afford an increased energy absorption [64].

The next step in this study was to explore two other types of aramidic fibers, TWARON T730 WRT (specific mass 260 g/m^2^) and Twaron LFT AT FLEX (specific mass 490 g/m^2^), and to combine these aramidic layers with STF, to investigate the possibility of improving the ballistic protection performances of the composite structures. The performed experimental tests started from a structure similar to that used in the manufacture of ballistic protection equipment (up to 20 layers). Details about the configurations tested are summarized in Table 3 and described below.

**Figure 10:**
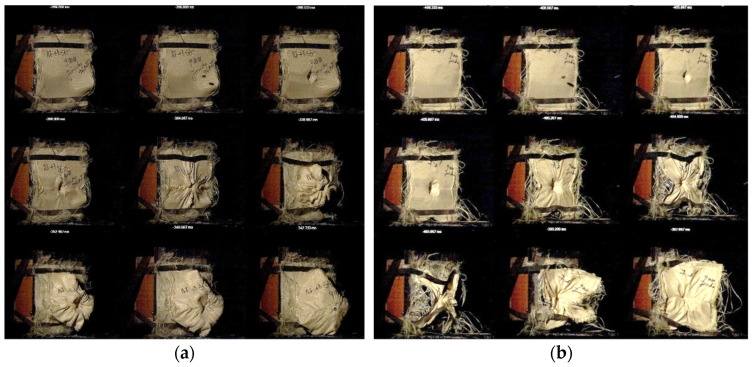
High-velocity impact behavior of (**a**) SC7 and (**b**) SC8.

The composite structure SC7 was subjected to high-velocity impact tests and the bullet was captured in the first five layers, while the depth of the traumatic imprint in the ballistic clay was measured at 19 mm (±1 mm). Figure 10a illustrates some images highlighting the impact behavior of the SC7 structure. Consequently, the number of Twaron T730 WRT layers was reduced until reaching a borderline behavior for structure SC8. When SC8 was subjected to high-velocity impact tests (Figure 10b), even if the bullet was retained in the ballistic package, the traumatic imprint was too cavernous (measuring approximately 70 mm in depth).

**Figure 11:**
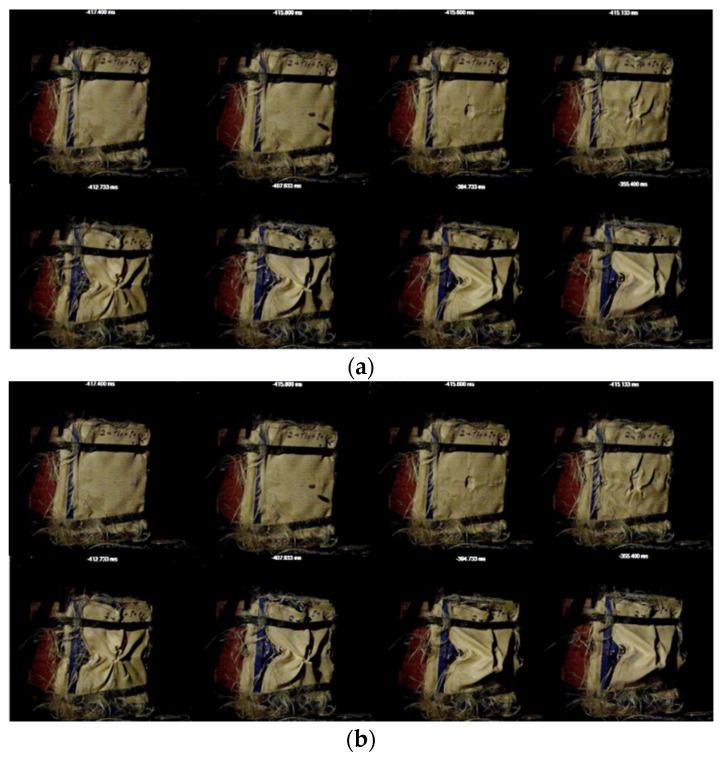
High-velocity impact behavior of (**a**) SC9 and (**b**) SC10.

The subsequent structures (SC9 and SC10, configurations detailed in Table 3, Figure S5h,i, and described below) were an attempt to evaluate the behavior of the P10 mixture in composite structures derived from the SC8 structure (which showed characteristics of borderline ballistic protection, as mentioned above). The SC9 structure (Figure 11a and Figure S5h) was obtained by overlapping two layers of Twaron T730 WRT, a ‘honeycomb 3D printed PLA structure’ filled with P10, and five layers of Twaron T730 WRT, followed by one last layer of Twaron LFT AT FLEX. The 9 mm bullet penetrated the structure; thus, the ‘honeycomb’ structure did not improve the ballistic protection characteristics. The SC10 structure (Figure 11b and Figure S5i) was obtained by placing P10 (polyethylene packaging, 2 cm thickness) between two layers of Twaron T730 WRT, five layers of Twaron T730 WRT, and one layer of Twaron LFT AT FLEX. The use of the P10 formulation did not enhance the protection level of the structure, because the bullet managed to perforate the composite structure.

Although the P10 shear thickening formulations did not lead to an increase in ballistic protection against high-velocity impact in either the honeycomb or homogenous configuration, this presents an opportunity to explore other potential solutions to enhance ballistic protection. Consequently, to increase protection and guarantee the highest level of safety, different approaches must be investigated and implemented only when the ballistic protection characteristics are in accordance with the requirements of NIJ standards [60]. The only non-Newtonian fluid formulations identified in this experimental investigation that improved the ballistic protective properties of composite structures (in dynamic loads at high speeds and collision with blunt items) included PEG 400 and 27–30 wt.% Aerosil (P4 and P10, respectively). It has been demonstrated that the arrangement of the STF between the aramid layers is crucial. Completely different results can be obtained using the same materials depending on the positioning of the STF layer, as was observed for the structures SC5 and SC6.

**Figure 12:**
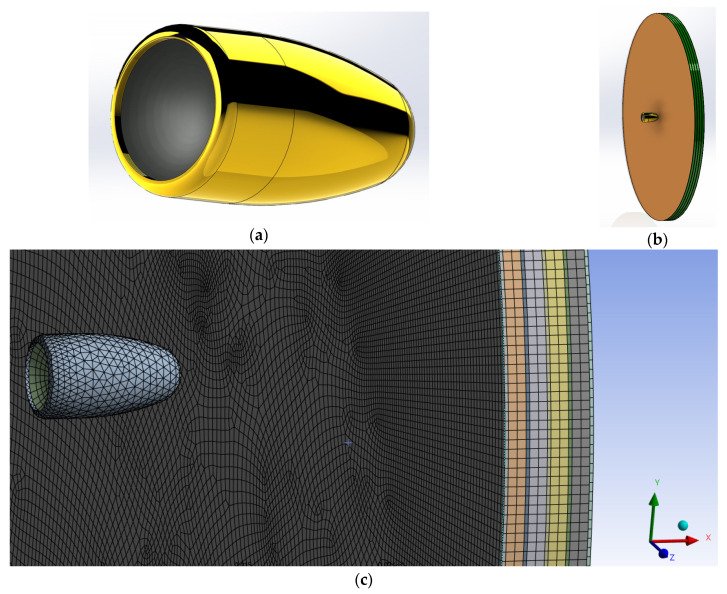
Model setup for numerical simulation.

**Figure 13:**
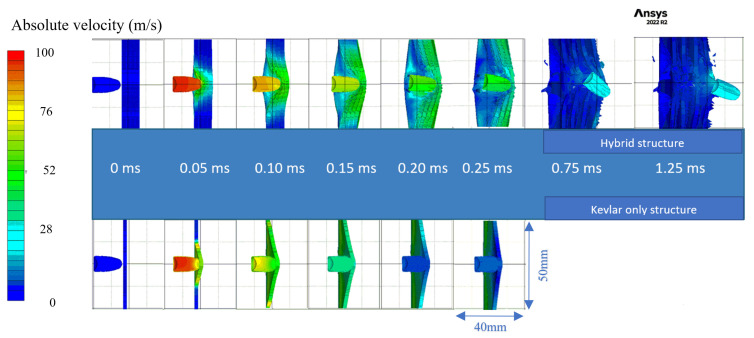
Velocity plot during impact.

**Figure 14:**
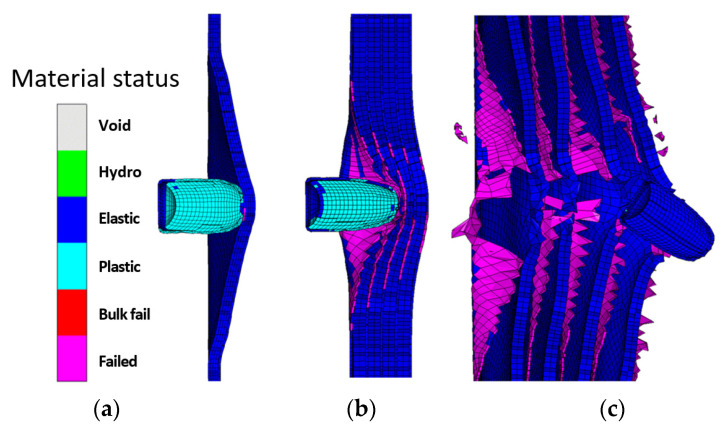
Material status plot: (**a**) at 0.1 ms after impact with Kevlar-only structure; (**b**) at 0.1 ms after impact with hybrid structure; (**c**) at 1 ms after impact with hybrid structure.

After conducting a thorough analysis of the dynamics of STF-based aramid composites, as the final phase of these investigations, their impact with a 9 mm bullet was modelled and simulated for a better understanding of the influence of the STF interlayer on the ballistic resistance of the Kevlar sheets. Explicit analysis was implemented, with a minimum 10^−11^–10^−8^ s timestep, automatically adjusted by the energy–velocity–deformation criterion. The Lagrange–Lagrange interaction was implemented to simulate the impact, with a 0.00028 mm contact gap condition, calculated automatically by the Autodyn software (Ansys® AutoDyn 2022 R2). The copper jacket and the lead core had the contact set to bonded–separable, while the Kevlar layers and the STF layers were set to contact each other in frictionless mode. The material models described in the supplementary data (Tables S1 and S2) were used from the Ansys explicit materials library, with the exception of the STF fluid, which material model was adapted by the authors by editing the parameters described in refs. [65,66,67]. The two sandwich structures had boundary conditions of fixed position for the radial surface of the disk. The bullet (Figure 12a) was positioned at 0.5 mm from the target (Figure 12b), and the initial velocity was set to 100 m/s. The model meshing was set to explicit linear with a maximum size of element of 1 mm, defeature size of 0.1 mm, and capture curvature option activated. The model mesh resulted in 846,313 nodes and 500,041 elements for the Kevlar/non-Newtonian fluid (KS) sandwich and 388,261 nodes and 196,777 elements for the Kevlar (K) sandwich (Figure 12c). The simulation showed that the interlayered structure does not retain the bullet but greatly reduces the bullet velocity and partially deforms it (Figure 13 and Figure 14). The jacket remains fully attached to the core and only exfoliates from the lead core at the tip of the projectile. When impacting the only-Kevlar sandwich structure, the bullet is fully retained in the “sandwich” structure and is repelled in the incoming direction.

**Figure 15:**
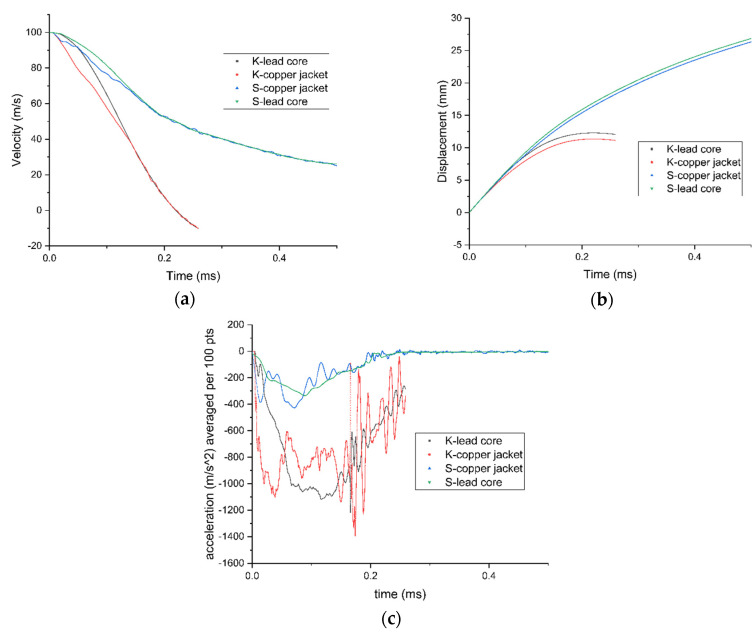
(**a**) Velocity profile in × direction (trajectory) for bullet components (jacket and core) for Kevlar-only structure (K) and hybrid sandwich structure (S); (**b**) Movement in x direction (trajectory) for bullet components (jacket and core) for Kevlar-only structure (K) and hybrid sandwich structure (S); (**c**) Acceleration in X direction (trajectory) of for bullet components (jacket and core) for Kevlar-only structure (K) and hybrid sandwich structure (S).

**Figure 16:**
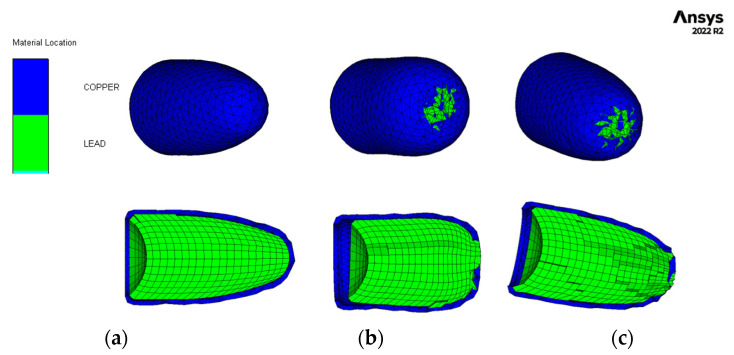
Material location plot for the projectile. (**a**) The initial aspect of the projectile; (**b**) Projectile after impact with Kevlar-only structure; (**c**) Projectile after impact with hybrid structure.

**Figure 17:**
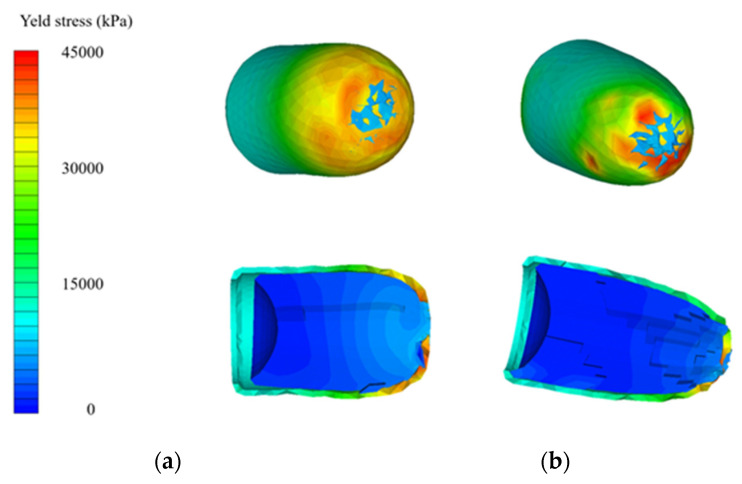
Yield stress plot. (**a**) After impact with Kevlar-only structure; (**b**) After impact with hybrid structure.

The bullet presents more consistent deformation while the jacket is partially detached from the core and the projectile has an exposed tip. In the Kevlar structure, the projectile suffers from a more prominent detachment of the jacket, which experiences a great loss of velocity right after initial impact. The lead core is slowed down in the Kevlar layers and is fully stopped at 0.21 ms. The negative velocity (Figure 15a) means that the bullet (jacket and core) starts moving backwards. The bullet that strikes the hybrid structure passes through the Kevlar/non-Newtonian fluid-layered structure as an unaltered element (without exhibiting a consistent jacket separation). The exit velocity is about 34 m/s, meaning that the projectile loses 88% of its kinetic energy due to impact. The movement graph (Figure 15b) shows that the projectile impacting the multilayered Kevlar is stopped in the structure and sent backwards due to the elastic response of the protection material. The maximum displacement of the bullet is 12 mm, 600% of the Kevlar multilayered thickness. When impacting the hybrid protection structure (Kevlar/non-Newtonian fluid alternation) the projectiles protrude through the material and continue to move in a xz direction, being diverted from the initial trajectory. The acceleration graph (Figure 15c) shows the higher stiffness of the Kevlar structure, the projectile being decelerated at a higher rate compared with when impacts the hybrid structure (STF interlayers). In the first phase of the impact, the jacket suffers high deceleration, compared to the lead core of the bullet, due to the high friction and flattening (Figure 16 and Figure 17) when impacting the first layer of Kevlar, both in the Kevlar-only and hybrid structure.

The simulations performed describe the unexpected detrimental effect of interlayering Kevlar with non-Newtonian fluid in terms of ballistic protection. In the only-Kevlar structure, the stress is transmitted in the whole structure and the deformation of the first layer is limited due to the response of the contacting layers. In this case, the deformation is distributed on a large surface and the layers act in the limit of elasticity. In the second case, the separation of the Kevlar layers with the non-Newtonian fluid causes a dramatic loss in local stiffness and the projectile impacts the Kevlar layers individually, one at a time, causing greater deformation in the impact zone over the elongation at break limit, causing the whole structure to progressively collapse and the projectile to penetrate. It could be presumed that the presence of the non-Newtonian fluid does not exhibit any favorable or detrimental effect on the impact phenomena. The change in the ballistic performance of the structure is mainly given by the layer distancing, thus generating an individual, progressive response in each layer. In other terms, in the case of air or any other soft material present between the Kevlar layers, the same response would be expected.

However, when P4 (PEG400, Aerosil 30 wt.%) was placed between layers no.1 and no.2 from a total of 11 layers of Kevlar XP, the bullet was stopped and entrapped in layer no.6 of the Kevlar XP, while the traumatic imprint was less profound than the reference sample (SC0—11 layers of Kevlar); thus, these positive results are worth further exploring, to better understand the influence of each variable on the dynamics of the ballistic protection effect.

This study provides crucial insights that can be used to optimize the performance of these materials in high-velocity impact scenarios. The information obtained within the confines of the experimental investigations performed can serve as a valuable foundation for future research and development in this field.

## 4. Conclusions

In this work, Kevlar^®^-based soft armor structures were investigated. The first stage of this study comprised the evaluation of the rheological properties of ten types of shear thickening formulations (STFs) to assess their possibility to be utilized as a component of “liquid body armor” for ballistic protection applications. Only the formulations containing PEG400 and 20–30 wt.% filler exhibited a shear thickening behavior. The evaluation of their performances during dynamic loading at moderate impact speed and their ability of kinetic energy absorption upon impact with standard shrapnel showed that some of these shear thickening fluids (STFs) are suitable for Kevlar^®^-based soft armor structures. Only P10 (PEG400, 27 wt.% Aerosil) exhibited a different response under dynamic loading, namely a significant increase in viscosity following dynamic impact with a free-falling blunt item. The kinetic energy absorption at impact with standard shrapnel tests revealed that the formulations ensured an energy absorption ranging between 45 to 55% (absorbed energy values up to 24 J) for a layer thickness of 35 mm. In contrast, the P10 formulation absorbed 60% of the kinetic energy (absorbed energy ~30 J) in the same testing conditions. The multilayer/composite structures were developed by considering all aspects of formulation production, rheological behavior analysis, dynamic impact behavior with blunt objects, and energy absorption at impact with standard shrapnel. The composite structures were further subjected to high-velocity impact tests which revealed that the distance between the aramid layers plays a crucial role in the ballistic response of Kevlar^®^–STF composite structures; otherwise, STF could even facilitate the penetration of Kevlar. The STFs’ energy absorption capacity relies on their viscosity variation upon a high shear rate, which causes high friction between the STF components due to intermolecular interactions and polymer chains’ entanglement. In contrast, the Kevlar fibers’ response is dependent on their packing/multilayer structure. When the STF is introduced between the Kevlar sheets, this packing is disrupted and the energy absorption capacity of the material is not improved.

The only formulation that led to a less profound traumatic imprint and stopped the bullet was P4 (PEG400, Aerosil 30 wt.%), when it was placed between layers no.1 and no.2 from a total of 11 layers of Kevlar XP. These experimental findings align with the modeling and simulation of Kevlar^®^–STF composites using Ansys simulation software (Ansys^®^ AutoDyn 2022 R2).

This work provides valuable insights for optimizing Kevlar interlayering with non-Newtonian shear thickening fluids for developing ballistic protection materials for high-velocity impact scenarios. Further research and development in this field may benefit from the information revealed in this study, achieved through specific ballistics tests, sustained by modeling and simulation of the phenomena.

## Figures and Tables

**Table 1:** STF formulations chosen for further experimental investigations.

Sample Code	Polymeric Matrix	Fumed Silica (0.2–0.3 μm) [wt.%]	Aerosil HDK^®^ N20 Pyrogenic Silica (>40 μm) [wt.%]
P1	PEG 400	20	-
P2	PEG 400	30	-
P3	PEG 400	-	20
P4	PEG 400	-	30
P5	PPG 400	20	-
P6	PPG 400	-	20
P7	PEG 400 *	-	40
P8	PEG 200 *	-	40
P9	PPG 400 *	-	40
P10	PEG 400	-	27
P-bk	PEG 400	-	-

* Ethyl alcohol was added to facilitate dispersion.

**Table 2:** Impregnated aramid–STF composites.

Impregnated Composite Sample Code	STF Code	Kevlar XP (No. of Layers)		Kevlar–Carbon Fiber (No. of Layers)		Specific Mass, Kg/m^2^
		Neat	Impregnated with STF	Neat	Impregnated with STF	
SC0	-	11	-	-	-	5.50
SC1	-	5	-	6	-	3.49
SC2	P1	5	6	-	-	8.23
SC3	P10	5	6	-	-	9.75
SC-bk	P-bk	5	6	-	-	8.77

**Table 3:** Multilayered aramid–STF composites.

Multilayered Composite Sample Code	STF Code	Kevlar XP (No. of Layers)		Twaron T730 WRT (No. of Layers)		Twaron LFT AT FLEX (No. of Layers)		Specific Mass, Kg/m^2^
		Neat	STF Packed Layer	Neat	STF Packed Layer	Neat	STF Packed Layer	
SC4	P2	11	1 (facing the bullet)	-	-	-	-	13.51
SC5	P4	11	1 (facing the bullet)	-	-	-	-	20.00
SC6	P4	11	1 (covered with one layer of Kevlar)					20.00
SC7	-	-	-	17	-	1	-	4.91
SC8	-	-	-	7	-	1	-	2.31
SC9	P10	-	-	7	1 (honeycomb structure)	1	-	16.48
SC10	P10	-	-	7	1 (rectangular structure)	1	-	16.48

**Table 4:** Velocities and kinetic energies measured for standard shrapnel impact test.

Sample Code	Projectile Initial Velocity, m/s	Initial Kinetic Energy, J	Projectile Residual Velocity, m/s	Residual Kinetic Energy, J	Absorbed Energy, J	Absorbed Energy, %
P5	269	39.80	179	17.62	22.18	55.73
P6	267	39.21	176	17.04	22.17	56.54
P7	312	53.54	229	28.84	24.70	46.13
P8	269	39.80	181	18.02	21.78	54.72
P9	285	44.67	211.4	24.58	20.09	44.97
P10	300	49.50	192	20.28	29.22	59.03

**Table 5:** Projectile initial velocity and kinetic energy.

Sample Code	Projectile Initial Velocity, m/s	Initial Kinetic Energy, J	Projectile Residual Velocity, m/s	Residual Kinetic Energy, J	Absorbed Energy, J	Absorbed Energy, %
SC0	429	741.7	N/A	N/A	741.7	100
SC1	428	738.2	234	220.7	517.5	70.1
SC2	432	752.1	204	167.7	584.4	77.7
SC3	430	745.1	178	127.7	617.4	82.9
SC4	433	755.6	198	158.0	597.6	79.1
SC5	438	773.1	169	115.1	658.0	85.1
SC6	431	748.6	N/A	N/A	748.6	100
SC7	435	762.6	N/A	N/A	762.6	100
SC8	433	755.6	N/A	N/A	755.6	100
SC9	434	759.1	N/A	N/A	759.1	100
SC10	432	752.1	N/A	N/A	752.1	100
SC-bk	428	738.2	266	285.7	452.5	61.3

## Data Availability

Data is contained within the article or Supplementary Material.

## Supplementary Materials

The following supporting information can be downloaded at: https://www.mdpi.com/article/10.3390/polym16162305/s1, Figure S1. Dynamic loading at a moderate impact speed; Figure S2. 3D printing of the casing system designed for STF loading; Figure S3. Experimental set-up for high-velocity impact tests; Figure S4. Aramidic fabrics utilized for high-velocity impact tests; Figure S5. Multilayered aramid. STF composites; Figure S6. 9 × 19 mm Parabellum bullet (Copper jacket and lead core); Table S1. Material models used in simulation; Table S2. Material models used in simulation.
