# Supplementary material for: Experimental Investigations on Shear Thickening Fluids as “Liquid Body Armors”: Non-Conventional Formulations for Ballistic Protection
Source: Polymers (Basel). 2024 Aug 15;16(16):2305. doi: 10.3390/polym16162305 (PMC11359824; doi:10.3390/polym16162305)
Supplement: Supplementary file 1 [file polymers-16-02305-s001.zip › polymers-3092250-supplementary.pdf]

polymers

# Supporting information

# Experimental Investigations on Shear Thickening Fluids as "Liquid Body Armors": Non-Conventional Formulations for Ballistic Protection

**Florentina Alexe [1,†], Ciprian Sau [1,†], Ovidiu Iorga [1,*], Gabriela Toader [2,*], Aurel Diacon [2,3], Edina Rusen [3], Claudiu Lazaroaie [1], Raluca Elena Ginghina [1], Tudor Viorel Tiganescu [2], Mircea Teodorescu [3] and Arcadie Sobetkii [4]**

[1] Research and Innovation Center for CBRN Defense and Ecology, 225 Olteniţei Ave., 041327 Bucharest, Romania
[2] Military Technical Academy "Ferdinand I", 39–49 George Cosbuc Boulevard, 050141 Bucharest, Romania
[3] Faculty of Chemical Engineering and Biotechnologies, National University of Science and Technology POLITECHNICA Bucharest, 1–7 Gh. Polizu Street, 011061 Bucharest, Romania
[4] SC MGM Star Construct SRL, 7 Pincota Street, 021784 Bucharest, Romania
* Correspondence: ovidiu.iorga@nbce.ro (O.I.); nitagabriela.t@gmail.com (G.T.)
† These authors contributed equally to this work.

## Contents

Figure S1 – Dynamic loading at a moderate impact speed

Figure S2 – 3D printing of the casing system designed for STF loading

Figure S3 – Experimental set-up for high-velocity impact tests

Figure S4 – Aramidic fabrics utilized for high-velocity impact tests

Figure S5 – Multilayered aramid – STF composites

Figure S6 – 9×19mm Parabellum bullet (Copper jacket and lead core)

Table S1 – Material models used in simulation

Table S2 – Material models used in simulation

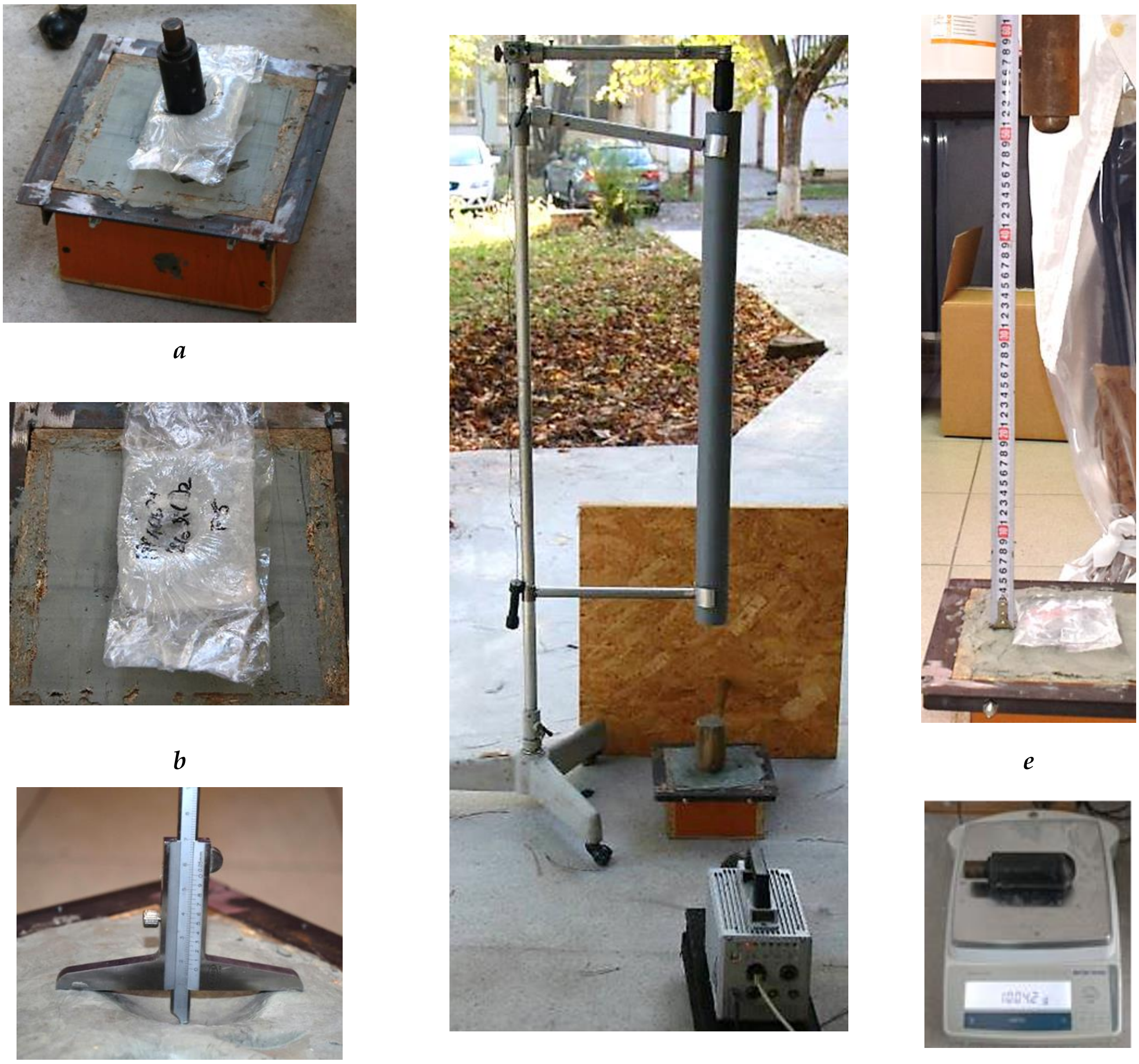

*a* *b* *c* *d* *e* *f*

| *g – Depth of the traumatic imprints measured in the ballistic clay* | | | | | |
|---|---|---|---|---|---|
| *Sample* | *Traumatic depth [mm]* | *Sample* | *Traumatic depth [mm]* | *Sample* | *Traumatic depth [mm]* |
| *P1* | *27 ± 2.1* | *P5* | *32 ± 2.7* | *P9* | *34 ± 1.5* |
| *P2* | *15 ± 1.3* | *P6* | *33 ± 1.9* | *P10* | *11 ± 1.2* |
| *P3* | *26 ± 2.0* | *P7* | *34 ± 1.3* | *P-bk* | *35 ± 1.3* |
| *P4* | *16 ± 1.5* | *P8* | *33 ± 2.4* | | |

## *Figure S1 – Dynamic loading at a moderate impact speed*

*(a) the capture of the impact of the blunt object with STF formulation; (b) the aspect of the STF after impact; (c) measuring the deformation depth in ballistic clay; (d) set-up design for the evaluation of dynamic loading at a moderate impact speed; (e) measuring the distance from the launching point to impact point; (f) aspect and mass of the blunt object utilized for this test set-up; (g) Depth of the traumatic imprints measured in the ballistic clay.*

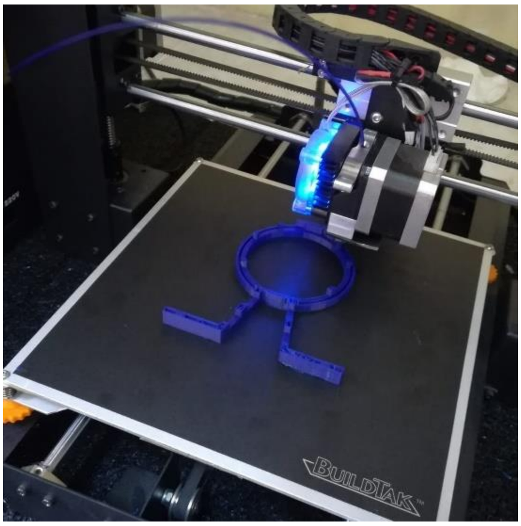


a

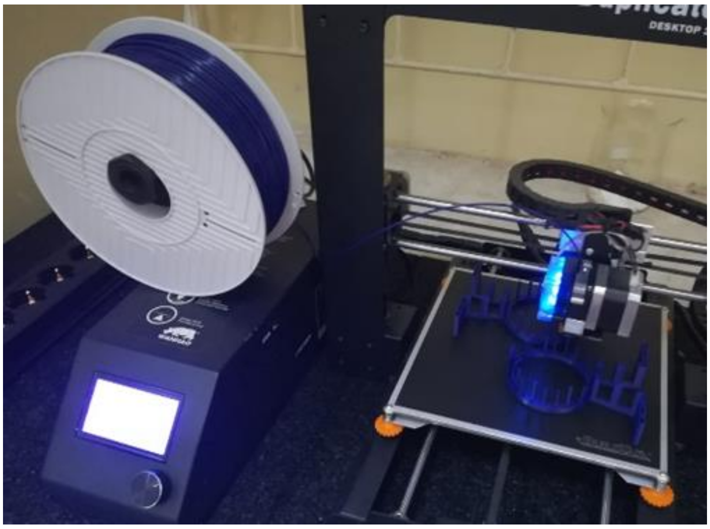

b

*Figure S2. – 3D printing of the casing system designed for STF loading.*

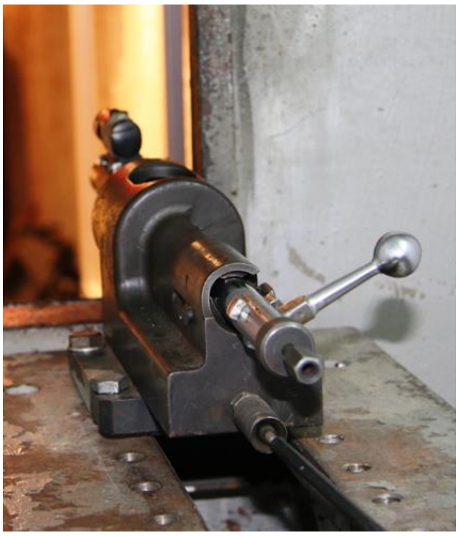

a

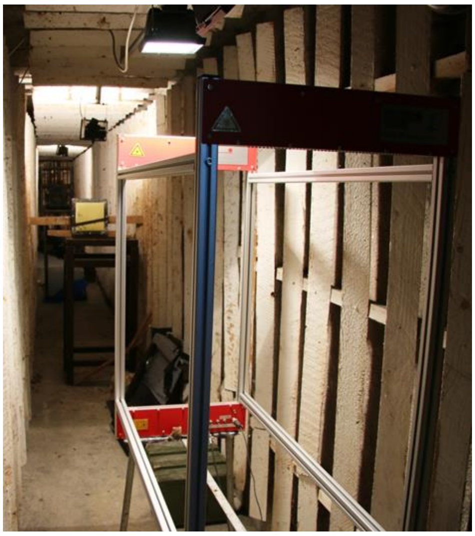

c

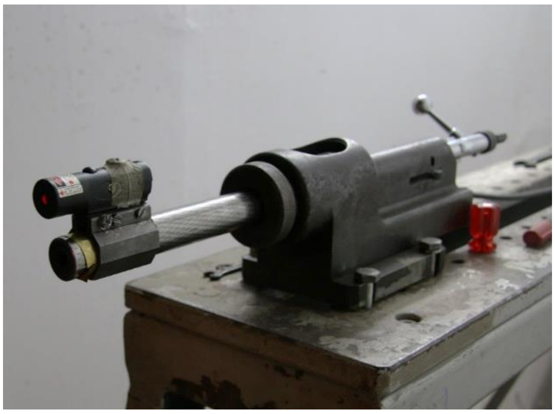

b

*Figure S3. – Experimental set-up for high-velocity impact tests.*
*(a,b) shooting stand; (c) ballistic chronograph*

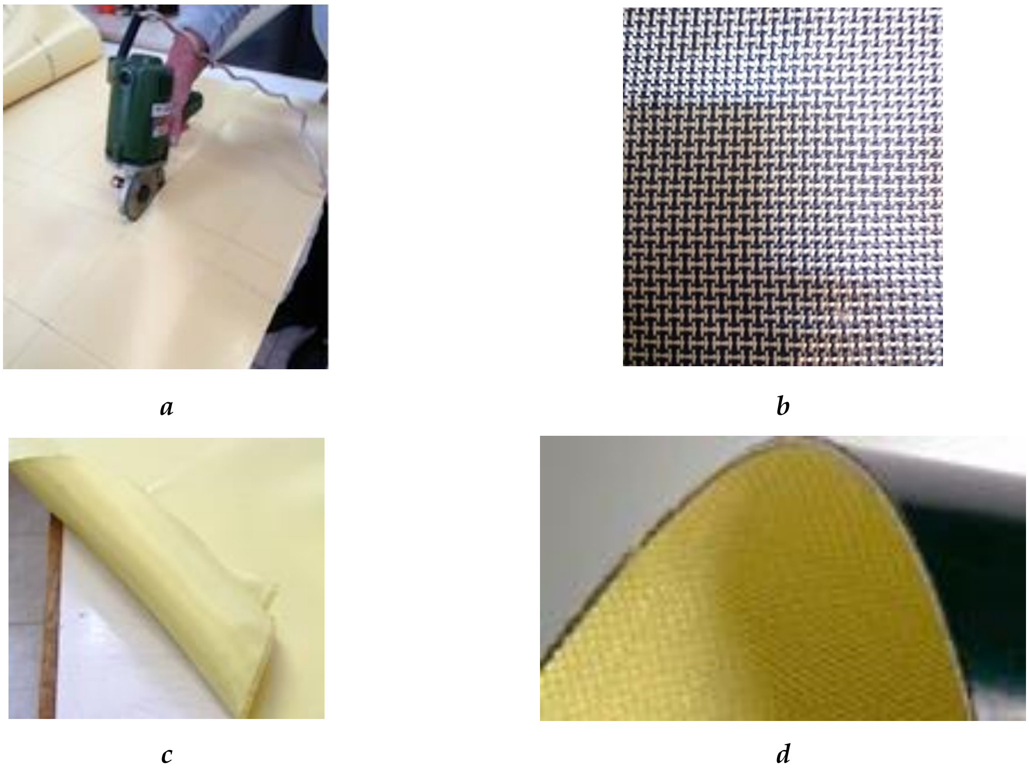


*Figure S4 – Aramidic fabrics utilized for high-velocity impact tests*

***(a) Kevlar XP; (b) Kevlar – carbon fiber composite fabric; (c) Twaron T730 WRT; (d) Twaron LFT AT FLEX***

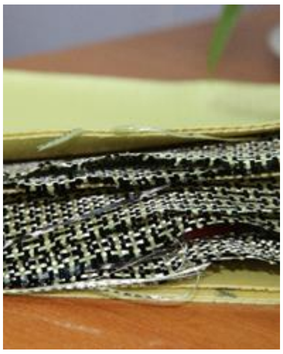

*a*

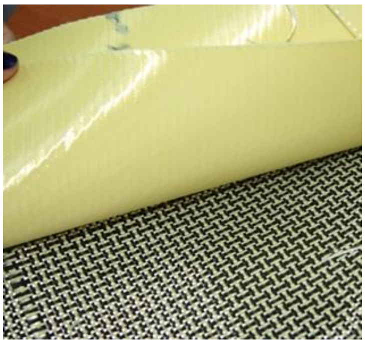

*b*

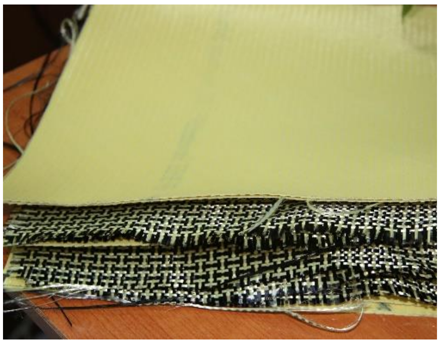

*c*

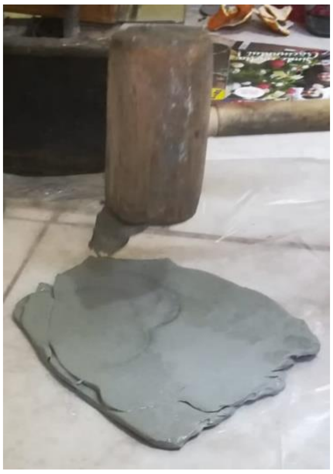

*d*

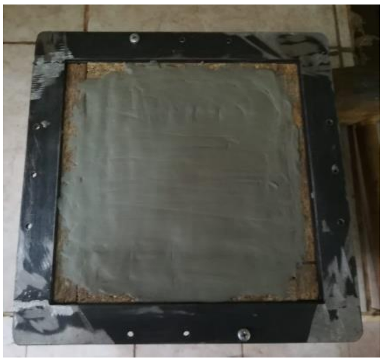

*e*

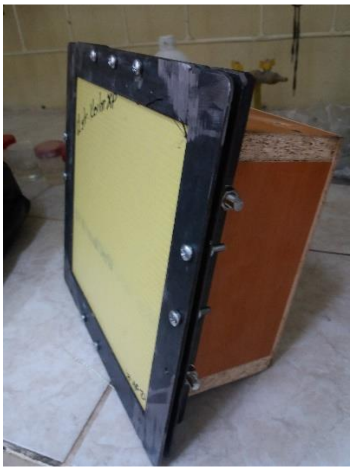

*f*

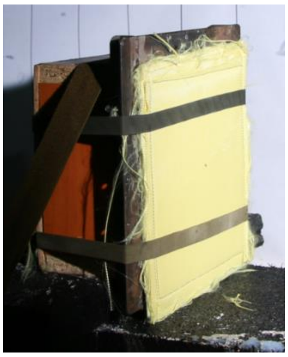

*g*

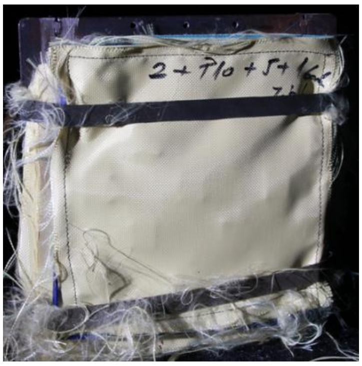

*h*

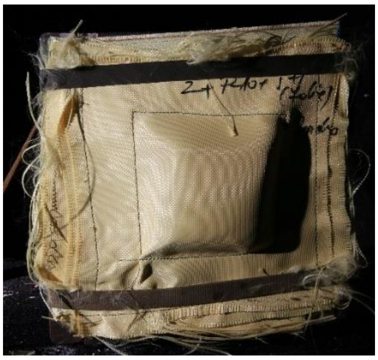

*i*

## *Figure S5 – Multilayered aramid – STF composites*

*(a,b,c) SC1; (d,e)ballistic clay; (f)SC0 placed over the ballistic clay tray; (g) SC8 placed over the ballistic clay tray before high velocity test; (h) SC9 placed over the ballistic clay tray before high velocity test; (i)SC10 placed over the ballistic clay tray before high velocity test;*

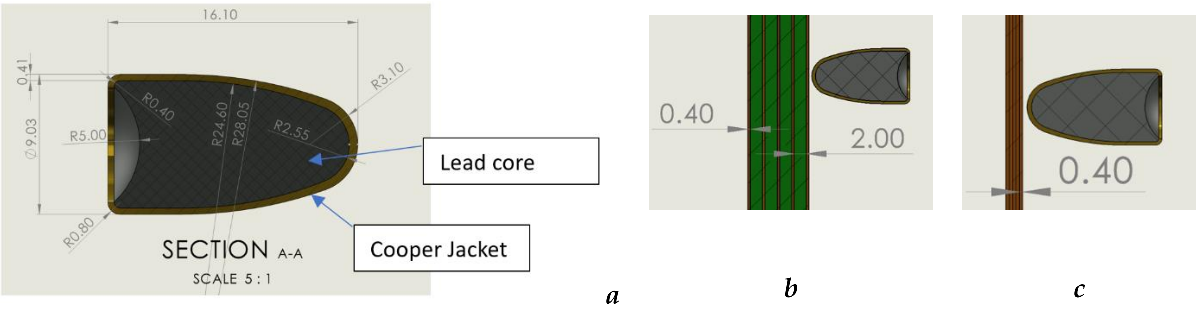


*a* *b* *c*

## *Figure S6 – 9×19mm Parabellum bullet (Copper jacket and lead core)*

***(a) 3D CAD model of the 9x19mm Parabellum bullet; The two impact models: (b) 5x Kevlar/4x non-Newtonian fluid alternating sandwich and (c) 5x Kevlar sandwich model***

## *Table S1 - Material models used in simulation*

| ***Part*** | ***Jacket*** | ***Core*** | ***Kevlar*** | ***Non-Newtonian fluid*** |
|---|---|---|---|---|
| ***Material type*** | Copper | Lead | Polyaramid | PEG/$SiO_2$ particles |
| ***Equation of state*** | Shock | Shock | Ortho | Linear |
| ***Strength model*** | Multilinear Hardening | Steinberg Guinan | Elastic | Elastic |
| ***Erosion model*** | None | None | None | Geometric Strain |
| ***Failure model*** | None | None | Material Stress/Strain | Principal Stress |
| ***Reference*** | - | - | [1] | [2] |

*Table S2 – Materials details for simulations*

# ANSYS AUTODYN

# Materials in model (click for shortcut)

| | | | | | |
|---|---|---|---|---|---|
| COPPER | LEAD | KEVLAR | NNF | - | - |

# Material Name - COPPER

| **Equation of State** | **Shock** |
|---|---|
| Reference density | 8.90000E+00 (g/cm3 ) |
| Gruneisen coefficient | 2.00000E+00 (none ) |
| Parameter C1 | 3.95800E+03 (m/s ) |
| Parameter S1 | 1.49700E+00 (none ) |
| Parameter Quadratic S2 | 0.00000E+00 (s/m ) |
| Relative volume, VE/V0 | 0.00000E+00 (none ) |
| Relative volume, VB/V0 | 0.00000E+00 (none ) |
| Parameter C2 | 0.00000E+00 (m/s ) |
| Parameter S2 | 0.00000E+00 (none ) |
| Reference Temperature | 2.95150E+02 (K ) |
| Specific Heat | 0.00000E+00 (J/kgK ) |
| Thermal Conductivity | 0.00000E+00 (J/mKs ) |
| **Strength** | **Multilinear Hardening** |
| Shear Modulus | 4.64000E+07 (kPa ) |
| Eff. Plastic Strain #1 | 0.00000E+00 (none ) |
| Eff. Plastic Strain #2 | 3.00000E-01 (none ) |

| | |
|---|---|
| Eff. Plastic Strain #3 | 1.00000E+20 (none ) |
| Eff. Plastic Strain #4 | 0.00000E+00 (none ) |
| Eff. Plastic Strain #5 | 0.00000E+00 (none ) |
| Eff. Plastic Strain #6 | 0.00000E+00 (none ) |
| Eff. Plastic Strain #7 | 0.00000E+00 (none ) |
| Eff. Plastic Strain #8 | 0.00000E+00 (none ) |
| Eff. Plastic Strain #9 | 0.00000E+00 (none ) |
| Eff. Plastic Strain #10 | 0.00000E+00 (none ) |
| Stress #1 | 1.20000E+05 (kPa ) |
| Stress #2 | 4.50000E+05 (kPa ) |
| Stress #3 | 4.50000E+05 (kPa ) |
| Stress #4 | 0.00000E+00 (kPa ) |
| Stress #5 | 0.00000E+00 (kPa ) |
| Stress #6 | 0.00000E+00 (kPa ) |
| Stress #7 | 0.00000E+00 (kPa ) |
| Stress #8 | 0.00000E+00 (kPa ) |
| Stress #9 | 0.00000E+00 (kPa ) |
| Stress #10 | 0.00000E+00 (kPa ) |
| Hardening | Isotropic |
| **Failure** | **None** |
| **Erosion** | **None** |
| **Material Cutoffs** | **-** |
| Maximum Expansion | 1.00000E-01 (none ) |
| Minimum Density Factor | 1.00000E-04 (none ) |
| Minimum Density Factor (SPH) | 2.00000E-01 (none ) |
| Maximum Density Factor (SPH) | 3.00000E+00 (none ) |
| Minimum Soundspeed | 1.00000E-06 (m/s ) |

| | |
|---|---|
| Maximum Soundspeed (SPH) | 1.01000E+20 (m/s ) |
| Maximum Temperature | 1.01000E+20 (K ) |
| **Reference:** | - |

# Material Name - LEAD

| **Equation of State** | **Shock** |
|---|---|
| Reference density | 1.13400E+01 (g/cm3 ) |
| Gruneisen coefficient | 2.74000E+00 (none ) |
| Parameter C1 | 2.00600E+03 (m/s ) |
| Parameter S1 | 1.42900E+00 (none ) |
| Parameter Quadratic S2 | 0.00000E+00 (s/m ) |
| Relative volume, VE/V0 | 0.00000E+00 (none ) |
| Relative volume, VB/V0 | 0.00000E+00 (none ) |
| Parameter C2 | 0.00000E+00 (m/s ) |
| Parameter S2 | 0.00000E+00 (none ) |
| Reference Temperature | 2.95150E+02 (K ) |
| Specific Heat | 1.24000E+02 (J/kgK ) |
| Thermal Conductivity | 0.00000E+00 (J/mKs ) |
| **Strength** | **Steinberg Guinan** |
| Shear Modulus | 8.60000E+06 (kPa ) |
| Yield Stress | 8.00000E+03 (kPa ) |
| Maximum Yield Stress | 1.00000E+05 (kPa ) |
| Hardening Constant | 1.10000E+02 (none ) |
| Hardening Exponent | 5.20000E-01 (none ) |
| Derivative dG/dP | 1.00000E+00 (none ) |
| Derivative dG/dT | -9.97600E+03 (kPa/K ) |

| | |
|---|---|
| Derivative dY/dP | 9.30400E-04 (none ) |
| Melting Temperature | 7.60000E+02 (K ) |
| **Failure** | **None** |
| **Erosion** | **None** |
| **Material Cutoffs** | - |
| Maximum Expansion | 1.00000E-01 (none ) |
| Minimum Density Factor | 1.00000E-04 (none ) |
| Minimum Density Factor (SPH) | 2.00000E-01 (none ) |
| Maximum Density Factor (SPH) | 3.00000E+00 (none ) |
| Minimum Soundspeed | 1.00000E-06 (m/s ) |
| Maximum Soundspeed (SPH) | 1.01000E+20 (m/s ) |
| Maximum Temperature | 1.01000E+20 (K ) |
| **Reference:** | - |

# Material Name - KEVLAR

| **Equation of State** | **Ortho** |
|---|---|
| Reference density | 1.65000E+00 (g/cm3 ) |
| Stiffness | Stiffness Matrix |
| C11 | 3.42500E+06 (kPa ) |
| C22 | 1.35000E+07 (kPa ) |
| C33 | 1.35000E+07 (kPa ) |
| C12 | 1.14000E+06 (kPa ) |
| C23 | 1.20000E+06 (kPa ) |
| C31 | 1.14000E+06 (kPa ) |
| Shear Modulus 12 | 1.00000E+06 (kPa ) |
| Shear Modulus 23 | 1.00000E+06 (kPa ) |

| | |
|---|---|
| Shear Modulus 31 | 1.00000E+06 (kPa ) |
| Material axes | X-Y-Z Space |
| Rotation angle about 11 (degrees) | 0.00000E+00 (none ) |
| X-coord. for dirn 11 (XYZ) | 0.00000E+00 (mm ) |
| Y-coord. for dirn 11 (XYZ) | 0.00000E+00 (mm ) |
| Z-coord. for dirn 11 (XYZ) | 1.00000E+00 (mm ) |
| Volumetric response | Polynomial |
| Bulk Modulus A1 | 4.15389E+06 (kPa ) |
| Parameter A2 | 4.00000E+07 (kPa ) |
| Parameter A3 | 0.00000E+00 (kPa ) |
| Parameter B0 | 0.00000E+00 (none ) |
| Parameter B1 | 0.00000E+00 (none ) |
| Parameter T1 | 4.15389E+06 (kPa) |
| Parameter T2 | 0.00000E+00 (kPa) |
| Reference Tempera-ture | 3.00000E+02 (K) |
| Specific Heat | 1.42000E+03 (J/kgK) |
| Thermal Conductiv-ity | 0.00000E+00 (J/mKs) |
| **Strength** | **Elastic** |
| Shear Modulus | 1.00000E+06 (kPa ) |
| **Failure** | **Material Stress/Strain** |
| Tensile Failure Stress 11 | 1.00000E+20 (kPa ) |
| Tensile Failure Stress 22 | 1.00000E+20 (kPa ) |

| | |
|---|---|
| Tensile Failure Stress 33 | 1.00000E+20 (kPa ) |
| Maximum Shear Stress 12 | 1.00000E+20 (kPa ) |
| Maximum Shear Stress 23 | 1.01000E+20 (kPa) |
| Maximum Shear Stress 31 | 1.01000E+20 (kPa) |
| Tensile Failure Strain 11 | 1.00000E-02 (none ) |
| Tensile Failure Strain 22 | 8.00000E-02 (none ) |
| Tensile Failure Strain 33 | 8.00000E-02 (none ) |
| Maximum Shear Strain 12 | 1.00000E+20 (none ) |
| Maximum Shear Strain 23 | 1.01000E+20 (none ) |
| Maximum Shear Strain 31 | 1.01000E+20 (none ) |
| Material Axes Option | IJK Space |
| Rotation angle about 11 (degrees) | 0.00000E+00 (none ) |
| Post Failure Option | Orthotropic |
| Residual Shear Stiffness Fraction | 2.00000E-01 (none ) |
| Maximum Residual Shear Stress | 1.00000E+20 (kPa ) |
| Decomposition Temperature | 7.00000E+02 (K ) |
| Matrix Melt Temperature | 1.01000E+20 (K ) |

| | |
|---|---|
| Failed in 11, Failure Mode | 11 only |
| Failed in 22, Failure Mode | 22 only |
| Failed in 33, Failure Mode | 33 only |
| Failed in 12, Failure Mode | 12 & 11 only |
| Failed in 23, Failure Mode | 23 & 11 only |
| Failed in 31, Failure Mode | 31 & 11 only |
| Melt Matrix Failure Mode | Bulk |
| Stochastic failure | No |
| **Erosion** | **None** |
| **Material Cutoffs** | - |
| Maximum Expansion | 1.00000E-01 (none ) |
| Minimum Density Factor | 1.00000E-05 (none ) |
| Minimum Density Factor (SPH) | 2.00000E-01 (none ) |
| Maximum Density Factor (SPH) | 3.00000E+00 (none ) |
| Minimum Sound-speed | 1.00000E-06 (m/s ) |
| Maximum Sound-speed (SPH) | 1.01000E+20 (m/s ) |
| Maximum Tempera-ture | 1.00000E+16 (K ) |
| **Reference:** | Hiermaier, Riedel, Hayhurst, Clegg, Wentzel - "Advanced Material Models for HVI Simulations" - E MI Report No. E43/99, ESA CR(P) 4305, 1999 |

# Material Name - NNF

| | |
|---|---|
| **Equation of State** | **Linear** |
| Reference density | 1.26500E+00 (g/cm3 ) |
| Bulk Modulus | 2.00000E+06 (kPa ) |
| Reference Temperature | 0.00000E+00 (K ) |
| Specific Heat | 0.00000E+00 (J/kgK ) |
| Thermal Conductivity | 0.00000E+00 (J/mKs ) |
| **Strength** | **Elastic** |
| Shear Modulus | 5.00000E+03 (kPa ) |
| **Failure** | **Principal Stress** |
| Principal Tensile Failure Stress | 3.45000E+04 (kPa ) |
| Max. Princ. Stress Difference / 2 | 1.01000E+20 (kPa ) |
| Crack Softening | No |
| Stochastic failure | No |
| **Erosion** | **Geometric Strain** |
| Erosion Strain | 4.00000E+00 (none ) |
| Type of Geometric Strain | Instantaneous |
| **Material Cutoffs** | - |
| Maximum Expansion | 1.00000E-01 (none ) |
| Minimum Density Factor | 1.00000E-04 (none ) |
| Minimum Density Factor (SPH) | 2.00000E-01 (none ) |
| Maximum Density Factor (SPH) | 3.00000E+00 (none ) |
| Minimum Soundspeed | 1.00000E-06 (m/s ) |

| | |
|---|---|
| Maximum Soundspeed (SPH) | 1.01000E+20 (m/s ) |
| Maximum Temperature | 1.01000E+20 (K ) |
| **Reference:** | W. Toqueboeuf et al. "Dynamic behaviour of Polycarbonate\polyurethane ..."-J.Phys IV France 7 19 97 |

[1] S. Hiermaier, W. Riedel, C. Hayhurst, R. Clegg, and C. Wentzel, "Advanced material models for hypervelocity impact simulations," *Contractor Report-European Space Agency CR P*, 2000.

[2] W. Toqueboeuf, B. Mortaigne, and C. Cottenot, "Dynamic behaviour of polycarbonate/polyurethane multi-layer for transparent armor," *Le Journal de Physique IV*, vol. 7, no. C3, pp. C3-499-C3-504, 1997.
